# What Patients With Asthma Share When No One Listens: Multimethod Observational Study of Patient Narratives on Reddit

**DOI:** 10.2196/77027

**Published:** 2026-01-08

**Authors:** Elena Curto-Sánchez, Gabriela Salazar-Palacios, Ana Martín-Varillas, Estela Cristina Prieto-Maíllo, Jacinto Ramos-González, Ignacio Dávila-González, Domingo Palacios-Ceña, Juan Nicolas Cuenca-Zaldivar

**Affiliations:** 1 Pneumology Service Complejo Hospitalario de Salamanca Salamanca, Castilla y León Spain; 2 Department of Allergy Institute for Biomedical Research of Salamanca Complejo Hospitalario de Salamanca Salamanca, Castilla y León Spain; 3 Research Group of Humanities and Qualitative Research in Health Science of Universidad Rey Juan Carlos (Hum&QRinHS) Department of Physical Therapy, Occupational Therapy, Physical Medicine and Rehabilitation Universidad Rey Juan Carlos Alcorcon, Madrid Spain; 4 Primary Health Center “El Abajón” Research Group of Pain and Physiotherapy Madrid Health Service Las Rozas de Madrid, Madrid Spain

**Keywords:** Reddit, subreddits, r/asthma, asthma, social media, health information, online health, online health information, patient education, observational study

## Abstract

**Background:**

The use of social media platforms, such as Reddit, to seek and share information about disease management and treatment strategies is increasingly common. In the context of asthma—a chronic condition characterized by limiting symptoms and exacerbations that require active patient engagement and adherence to treatment—there is a lack of research describing the content of Reddit posts and the specific topics of interest to patients.

**Objective:**

This study aimed to describe the topics discussed by users on the Reddit asthma forum and identify the sentiments and polarity of the language used in the posts.

**Methods:**

A retrospective observational study of public posts on the asthma subreddit forum (r/Asthma) over a 1-year period (October 2023-October 2024). All posts and related threads were included, subdivided into hot, news, and top, and those voted “up” or “down,” those that received “awards,” categorized as “golds.” The messages were reviewed manually and excluded if they were not related to asthma. A mixed methods analysis was conducted, comprising (1) analysis using text lemmatization, (2) structural topic modeling to identify topics based on word frequency, and (3) sentiment and polarity analysis. This approach aimed to identify the most frequently used topics on Reddit, detect positive and negative sentiments based on the words used, and acceptance or rejection (polarity) based on the language used in the asthma subreddit. Statistical analyses were performed using R software (version 4.1.3; R Foundation for Statistical Computing), with a significance threshold set at *P*<.05.

**Results:**

After removing duplicates, 7806 posts were identified. The suitability of the chosen analysis model was confirmed, as it presented the best balance between exclusivity and semantic coherence. Clusters of 25 topics were identified and distributed according to their weight. The topics with the highest weight were Topic 7 (Symptoms and severity of asthma attacks) and Topic 18 (Causes of asthma). No significant differences were found in the evolution of emerging topics throughout the year except in Topic 20 (Seeking advice from people with asthma; *P*=.04), Topic 21 (Medical tests that should be reviewed periodically; *P*=.04), and Topic 22 (Times of year when attacks occur; *P*=.03). The proportion of feelings and emotions showed a stable trend throughout the year. Discrepancies in feelings and emotions were identified depending on the dictionaries used. Thus, a higher probability of positive feelings was confirmed in the AFINN lexicon. Meanwhile, negative feelings were significant in the Stanford Natural Language Processing, Bing, and National Research Council Canada lexicons.

**Conclusions:**

These results can serve as a guide to identify hidden patient needs and help professionals develop specific interventions on topics relevant to patients.

## Introduction

Asthma is one of the most prevalent chronic respiratory diseases, affecting over 300 million people globally [1]. The intermittent onset of bronchospasm attacks causes symptoms such as wheezing and dyspnea, and is characterized by airway inflammation, hyperresponsiveness, and mucus hypersecretion, leading to airflow obstruction [1]. The clinical manifestation of asthma varies across individuals, with distinct phenotypes [2], influencing treatment of the disease [3].

The introduction of novel clinical biomarkers (such as sputum and blood eosinophils, serum immunoglobulin E (IgE), and fractional exhaled nitric oxide) [2] has facilitated the development of treatments tailored to each patient’s individual phenotype [4,5], specifically monoclonal antibodies. However, these drugs are limited to the most severe forms and certain phenotypes [3,4]. Treatment for most patients with mild to moderate forms of the disease is based on the use of inhalers; however, despite the ongoing development of educational programs, significant issues remain in relation to adherence [6] and inhalation technique [7], both of which have critical implications for disease management and the frequency of exacerbations.

Another key aspect in the treatment of asthma is the shared perspective between prescribing physicians and patients diagnosed with asthma on the impact of the disease on daily life [8,9]. Previous studies [8,9] show that there are differences in perspective on the management and control of the disease, resulting in an underestimation of the impact of asthma on the patient’s life by the physician, reducing disease and symptom control [8].

One of the responses among patients is to seek help to cope with symptoms, apply strategies, and share their experiences with other patients [10,11]. In this regard, the use of social media for health reasons has increased over the last decade [10,11], having a major impact on patients’ understanding of the disease, the acquisition of habits, the decision-making process, and health outcomes [12-18]. In addition, widely shared or viral content significantly influences the adoption—or rejection—of health behaviors [10], largely due to its ability to elicit strong, activating emotions, whether positive or negative [19].

Reddit is a large-scale online platform that hosts more than 130,000 individual communities, known as subreddits. It attracts around 500 million visits per month and engages approximately 73 million unique users each day [20]. With over 270 million active users each month [21], Reddit allows individuals to share personal experiences related to illness and health care. Users frequently post firsthand accounts, respond to others with shared experiences, and exchange advice on symptoms, medical conditions, and treatments [10,22]. The platform allows registered users (known as Redditors) to post content (text, images, and videos) to moderated boards, which are voted on by other users. Posts on Reddit are visible to the broader community, and their prominence within a subreddit is shaped by user voting—either upvotes or downvotes [23]. Subreddits are organized by topic, and users, known as Redditors, can subscribe to those that match their interests, allowing them to curate the content displayed on their personalized front page [24]. Additionally, some subreddits follow a question-and-answer format, with popular examples including r/AskReddit and r/AMA, which features the interactive “Ask Me Anything” style. Discussions on Reddit are public (unless specifically designated as private), allowing for passive data collection. Furthermore, for an internet user seeking information on a specific topic, Reddit is a useful starting point due to its topic-centric organization [10].

To the authors’ knowledge, there are no studies that have shown the use of Reddit by patients with asthma and described the topics they consult. The questions that guided this study were: “What were the most consulted and discussed topics by patients with asthma on Reddit?” and “Was the language used on Reddit positive and negative? The objectives of this study were (1) to describe the topics discussed by patients in the asthma subreddit (r/Asthma) and (2) to identify the sentiments and polarity of the language used by patients with asthma on Reddit.

This study may help identify unknown and unmet needs of patients with asthma and improve medical care.

## Methods

### Design

An observational study was conducted, consisting of a retrospective analysis of public posts on Reddit over a 1-year period, from October 2023 to October 2024. Also, we used the subreddit forum asthma (r/Asthma) [25]. This study adhered to the Reporting Guidelines for Social Networks in Health Research [26], which are incorporated within the EQUATOR (Enhancing the Quality and Transparency of Health Research) initiative [27].

### Data Collection

Posts that included general comments were identified and subdivided into hot, news, and top. Similarly, posts that were voted “up” or “down” were identified, as well as those that received “awards,” categorized as “golds,” and cross-posted posts. r/Asthma posts were obtained with the R library *RedditExtractoR* [28]. This library allows for extracting up to 1000 main posts by each category (hot, top, and new posts) and all generated threads. Only posts in the English language were used to facilitate the analysis.

All posts from the r/Asthma forum and all threads derived from the conversations were included, as r/Asthma is a subreddit forum dedicated specifically to asthma, so the objective of the study is to determine what topics interest the users of this forum without establishing any specific selection criteria a priori.

In addition, the main topic, secondary topics, number of votes obtained, number of responses, and subresponses obtained were included. Also, all information related to asthma, such as symptom management, treatment, follow-up consultations, risk behaviors, healthy habits, professional care, adherence (or nonadherence) to treatment and medical recommendations, education, use of inhalers, etc, was incorporated into the study and analyzed. Messages were reviewed manually and systematically excluded if they were not related to asthma use.

### Statistical Analysis

#### Overview

Statistical analyses were performed using R software (version 4.1.3; R Foundation for Statistical Computing) [29]. The significance level was set at *P*<.05. The Kolmogorov-Smirnov test with Lilliefors correction was used to test the distribution of the variables. The variables were described with mean (SD), median (IQR), or with absolute and relative values, that is, n (%).

In this study, a mixed methods analysis was carried out that included three types of analysis: (1) text lemmatization analysis, (2) structural analysis using models based on topics identified by their frequency of appearance, and (3) sentiment and polarity analysis. This allowed us to identify the most frequently used topics on Reddit, identify positive and negative sentiments based on the words used, and determine acceptance or rejection (polarity) based on the language used to discuss asthma in Reddit posts.

#### Analysis Using Text Lemmatization

First, the text of the posts was lemmatized for analysis. Lemmatization is a linguistic process that involves identifying the base or lexicon form of a word—known as the lemma—from its inflected variants (eg, plural forms, gendered adjectives, verb conjugations). The lemma is the canonical form under which all inflected versions of a word are grouped. For example, in traditional dictionaries, nouns are typically listed in their singular form, adjectives in the masculine singular, and verbs in the infinitive.

#### Structural Topic Modeling Analysis

Second, a structural topic model analysis was conducted to examine the emergence of topics across user comments. This method also allows the incorporation of metadata, such as the time period of each comment, as a covariate in the model. The selection of the optimal number of topics was carried out in 2 phases: first, the range of topics with the best values of held-out likelihood and lower limit of stability of convergence between iterations of the models and with the lowest value in the residuals was evaluated; in a second phase, the ratio between semantic coherence and exclusivity between the selected models was analyzed. Exclusivity evaluates whether the main words of the topics also appear as main words of other topics, while semantic coherence shows whether the words most associated with a topic occur equally or not within the documents; in both cases, higher values are better [30-32].

#### Sentiment and Polarity Analysis

Finally, a sentiment analysis was performed using the Bing [33], AFINN [34], National Research Council Canada (NRC) [35], and Stanford Natural Language Processing (Stanford NLP) [36] dictionaries. All 4 dictionaries are based on unigrams or individual words that assign scores for positive or negative feelings. In addition, the NRC lexicon classifies words into 8 emotional categories of anger, anticipation, disgust, fear, joy, sadness, surprise, and trust. While the Affin lexicon assigns words a score ranging from –5 to 5, with negative values indicating more negative feelings and positive values indicating more positive feelings. Stanford NLP classifies positive into 5 categories: very negative, negative, neutral, positive, and very positive. Similarly, sentence polarity was analyzed using the Bing lexicon, incorporating modifiers such as amplifiers, decrementers, and negators as proposed by Halliday [37]. The integration of these 4 dictionaries facilitates a detailed and comprehensive analysis of the emotions and feelings conveyed in the texts shared by Reddit (r/Asthma forum) users. This methodological approach enables an emotional profiling of the analyzed content, contributing to a deeper understanding of the affective dimensions embedded in user-generated discourse. Furthermore, this combination of dictionaries allows for a multiangle analysis, providing a comprehensive analytical framework that encompasses the analysis of positive and negative emotions, ranging from –5 to 5 with the AFINN dictionary or from very negative to very positive with the Stanford NLP dictionary, as well as assessing 8 more complex emotions with the NRC dictionary. Moreover, the simultaneous use of these 4 dictionaries allows for a cross-validation and complementarity strategy; the combination of results can lead to a more robust and reliable analysis [38,39].

The presence of significant differences in the proportion of the categories of feelings and emotions or the average score of each lexicon and its temporal evolution was analyzed using a regression model. The assumption of linearity between the dependent variables and the quantitative predictors was tested by evaluating the effective degrees of freedom (EDF) with a value greater than 1 as a nonlinearity cut-off point. A generalized additive mixed model (GAMM) or a linear mixed model (LMM) was applied depending on the EDF value. Since these were time series, the correlation structure was modeled by comparing, using a likelihood ratio test (LRT), the models without this structure, with an autoregressive structure with lag 1 (AR1), and with an autoregressive moving average structure (ARMA) evaluated by means of an ARMA analysis of the residuals of the previous AR1 model. In the GAMM models, the fulfillment of the assumptions of *v* for the smoothed terms was tested, eliminating those with a value greater than 0.8, and the adequacy of the number of basic functions was checked using the K index (*P*>.05 as a selection criterion). The fulfillment of the assumptions in the residuals was checked with the Kolmogorov-Smirnov tests with Lilliefors correction (normality) and Breusch-Pagan (heteroscedasticity). Since these were not met in the GAMM models, the robust sandwich-type standard errors were calculated in the LMM model with the Stanford NLP lexicon using a bootstrap.

### Ethical Considerations

This study does not require approval from the Ethics and Clinical Research Committee of the University Hospital of Salamanca because the information is publicly available. The same considerations have been applied in previous studies that analyzed the use of Reddit by patients to share and search for information on topics related to health and disease [40-42]. In addition, institutional review board and research ethics committee approvals were not required nor sought because the research did not meet the requirements for needing ethical approval per section 1.3 of the European Pharmaceutical Market Research Association guidelines [43]. The ethical principles established by the Declaration of Helsinki were respected. In addition, Reddit offers a comprehensive application programming interface that grants access to much of the platform’s information. In strict compliance with the rules and ethical guidelines of each subreddit, we only collect data from subreddits that explicitly allow research activities. Furthermore, to protect user privacy, we only record the comment ID without including any user information or the content of the comment itself, ensuring that no personally identifiable information or data that could lead to reidentification is collected. Furthermore, in the posts selected as illustrative examples for this study, all identifying information, including authorship and publication dates, was removed, and the content was summarized to preserve and emphasize the essential information.

## Results

### Descriptive

A total of 7806 posts were collected from the r/Asthma thread between October 2023 and October 2024 after removing duplicates, of which 454 generated a total of 6046 response threads. Since Reddit does not allow the extraction of more than 1000 posts, different filters were used to maximize the search for primary comments, from which an unlimited number of response threads could be obtained (Table 1).

**Table 1:** Reddit r/Asthma posts’ characteristics between October 2023 and October 2024.

Post characteristics				2023							2024																					
				October		November		December		January			February		March		April		May		June		July		August		September		October			
**Characteristics of posts**																																
	n			4		69		62		74			69		89		81		76		86		79		85		451		535			
	Generated comments, mean (SD)			37.75(10.24)		23.03 (19.39)		20.85 (15.28)		23.16 (24.78)			32.83 (31.77)		30.66 (23.66)		26.83 (27.63)		24.22 (19.82)		25.69 (23.05)		20.71 (20.76)		19.44 (26.46)		10.39 (14.26)		10.66 (13.71)			
	**Comments filter, n (%)**																															
		Hot^a^	—^b^		—		—		—			—		—		—		—		—		—		10 (11.8)		368 (81.6)		382 (71.4)				
		New^c^	—		—		—		—			—		—		—		—		—		—		2 (2.4)		—		—				
		Top^d^	4 (100)		69 (100)		62 (100)		74 (100)			69 (100)		89 (100)		81 (100)		76 (100)		86 (100)		79 (100)		73 (85.9)		83 (18.4)		153 (28.6)				
**Characteristics of posts with threads**																																
	n			1		13		12		26			22		28		28		27		18		17		28		132		102			
	Score^e^, mean (SD)			17.00		15.31 (5.89)		9.75 (2.05)		9.38 (3.73)			9.68 (4.75)		10.54 (6.13)		9.93 (5.28)		12.96 (7.60)		11.00 (4.00)		10.47 (3.74)		7.57 (8.16)		2.42 (3.57)		4.06 (5.68)			
	Upvotes^f^, mean (SD)			17.00		15.31 (5.89)		9.75 (2.05)		9.38 (3.73)			9.68 (4.75)		10.54 (6.13)		9.93 (5.28)		12.96 (7.60)		11.00 (4.00)		10.47 (3.74)		7.57 (8.16)		2.42 (3.57)		4.06 (5.68)			
	Up ratio^g^, mean (SD)			0.95		0.92 (0.07)		0.92 (0.07)		0.92 (0.07)			0.91 (0.09)		0.91 (0.06)		0.93 (0.08)		0.92 (0.07)		0.94 (0.07)		0.85 (0.11)		0.87 (0.15)		0.77 (0.24)		0.79 (0.24)			
	Cross-posts^h^, mean (SD)			—		—		—		—			—		—		—		—		—		—		—		0.01 (0.09)		—			
	Generated comments, mean (SD)			34.00		26.92 (18.83)		19.92 (14.15)		16.38 (12.54)			18.14 (15.79)		19.25 (14.04)		16.07 (11.69)		19.63 (15.80)		16.72 (16.05)		24.88 (24.83)		12.57 (9.80)		8.19 (8.56)		9.29 (14.69)			
**Thread characteristics**																																
	n			26		339		218		415			372		536		450		503		320		427		359		1099		982			
	Score, mean (SD)			1.77 (1.24)		2.99 (3.81)		2.56 (3.01)		2.87 (3.60)			2.92 (5.49)		2.25 (3.03)		2.85 (4.23)		2.41 (3.20)		2.83 (4.21)		3.02 (6.12)		2.72 (3.36)		2.19 (3.43)		2.49 (2.94)			
	Upvotes, mean (SD)			1.77 (1.24)		2.99 (3.81)		2.56 (3.01)		2.87 (3.60)			2.92 (5.49)		2.25 (3.03)		2.85 (4.23)		2.41 (3.20)		2.83 (4.21		3.02 (6.12)		2.72 (3.36)		2.19 (3.43)		2.49 (2.94)			

^a^Hot: Comments with the highest popularity rate based on their age and number of positive votes are the most active and popular discussions in real time.

^b^Not available.

^c^New: New comments generated in a given period of time.

^d^Top: Comments with the highest number of net upvotes (upvotes minus downvotes) in a given time period.

^e^Score: Net positive votes (positive votes minus negative votes).

^f^Upvotes: Positive votes.

^g^Up ratio: Quotient of positive and negative votes in percentage.

^h^Cross-posts: Comments linked from other subreddits. Generated comments: comments not written by humans.

### Analysis of Thematic Models

By determining the best held-out likelihood values among the models, it was determined that the optimal number of topics was 15 and 25 (Multimedia Appendix 1). Subsequently, the model with 25 topics was found to have the best balance of exclusivity versus* *semantic coherence, and therefore, the model with 25 topics was adopted (Multimedia Appendix 2). Table 2 shows the 25 topics hypothesized after the development of the model.

**Table 2:** Reddit r/Asthma posts topics identified.

Topic	Description
Topic 1	Influence of respiratory problems on the ability to walk.
Topic 2	Timing of attacks.
Topic 3	Hopes for rapid improvement.
Topic 4	Adverse effects of Prednisone that may justify stopping its use.
Topic 5	Gratitude for improvement.
Topic 6	Use of inhaled steroids as rescue medication.
Topic 7	Symptoms and severity of asthma attacks.
Topic 8	Medical tests to be monitored.
Topic 9	Influence of respiratory problems on work activity.
Topic 10	Influence of respiratory problems on quality of life in general terms.
Topic 11	Influence of tobacco.
Topic 12	Need for information.
Topic 13	Budesonide spray dosage.
Topic 14	How to access Albuterol inhalers for wheezing without medical insurance.
Topic 15	Hospitalization with oxygen therapy in case of acute exacerbation.
Topic 16	Search for any asthma-related therapy.
Topic 17	Influence of respiratory symptoms such as coughing.
Topic 18	Causes of asthma.
Topic 19	Symptom change throughout the year.
Topic 20	Request for advice to people who have asthma.
Topic 21	Medical tests that should be checked periodically.
Topic 22	Times of the year when attacks occur.
Topic 23	Change in symptoms at times of the year when temperatures are cooler.
Topic 24	Need for specialized medical care.
Topic 25	Gratitude for the assistance received.

**Figure 1:**
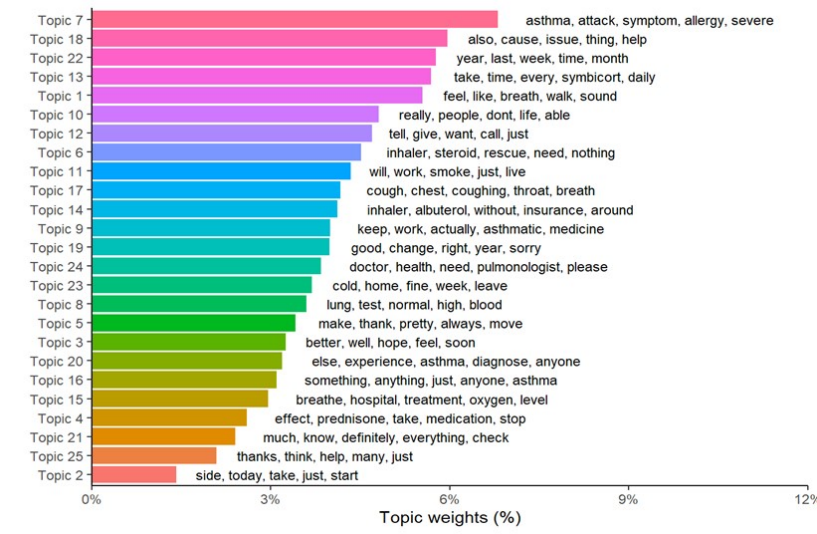
Weighted topics.

Subsequently, each topic was classified according to the weight of its frequency, together with the most frequent words used in each topic (Figure 1 shows the weighted topics).

The topics with the greatest weight were Topic 7 (Symptoms and severity of asthma attacks) and Topic 18 (Causes of asthma).

Regarding the annual analysis of the topics, there were no significant differences in the evolution of the emerging topics throughout the year, except in 3 topics. These were Topic 20 (Request for advice from people with asthma; *P*=.04), Topic 21 (Medical tests that should be checked periodically; *P*=.04), and Topic 22 (Times of the year when attacks occur; *P*=.03; Table 3).

**Table 3:** Evolution of each topic throughout the period of time analyzed.

Topic and time period			Coefficient (SE)		*t* statistic		*P* value^a^
**Topic 1**							
	Intercept	–0.04 (0.29)		–0.13		.89	
	Date	0 (0)		0.29		.76	
**Topic 2**							
	Intercept	–0.04 (0.21)		–0.18		.85	
	Date	0 (0)		0.30		.76	
**Topic 3**							
	Intercept	0.415 (0.21)		1.92		.05	
	Date	0 (0)		–1.77		.07	
**Topic 4**							
	Intercept	0.052 (0.24)		0.21		.82	
	Date	0 (0)		–0.07		.94	
**Topic 5**							
	Intercept	0.225 (0.21)		1.04		.29	
	Date	0 (0)		–0.89		.37	
**Topic 6**							
	Intercept	0.063 (0.26)		0.23		.81	
	Date	0 (0)		–0.07		.94	
**Topic 7**							
	Intercept	0.128 (0.33)		0.38		.70	
	Date	0 (0)		–0.22		.82	
**Topic 8**							
	Intercept	–0.234 (0.27)		–0.85		.39	
	Date	0 (0)		0.99		.32	
**Topic 9**							
	Intercept	0.308 (0.29)		1.05		.29	
	Date	0 (0)		–0.90		.36	
**Topic 10**							
	Intercept	0.142 (0.268)		0.53		.59	
	Date	0 (0)		–0.35		.72	
**Topic 11**							
	Intercept	0.089 (0.24)		0.37		.71	
	Date	0 (0)		–0.20		.83	
**Topic 12**							
	Intercept	0.053 (0.25)		0.20		.83	
	Date	0 (0)		–0.03		.97	
**Topic 13**							
	Intercept	–0.456 (0.30)		–1.50		.13	
	Date	0 (0)		1.66		.09	
**Topic 14**							
	Intercept	–0.452 (0.28)		–1.59		.11	
	Date	0 (0)		1.74		.08	
**Topic 15**							
	Intercept	–0.074 (0.31)		–0.23		.81	
	Date	0 (0)		0.36		.71	
**Topic 16**							
	Intercept	–0.432 (0.27)		–1.57		.11	
	Date	0 (0)		1.71		.08	
**Topic 17**							
	Intercept	–0.048 (0.23)		–0.20		.83	
	Date	0 (0)		0.37		.70	
**Topic 18**							
	Intercept	0.223 (0.31)		0.70		.48	
	Date	0 (0)		–0.53		.59	
**Topic 19**							
	Intercept	0.41 (0.24)		1.65		.09	
	Date	0 (0)		–1.48		.13	
**Topic 20**							
	Intercept	–0.505 (0.26)		–1.89		.05	
	Date	0 (0)		2.04		.041	
**Topic 21**							
	Intercept	0.421 (0.19)		2.11		.035	
	Date	0 (0)		–1.98		.048^b^	
**Topic 22**							
	Intercept	0.706 (0.30)		2.31		.021	
	Date	0 (0)		–2.13		.033	
**Topic 23**							
	Intercept	0.167 (0.31)		0.53		.59	
	Date	0 (0)		–0.39		.69	
**Topic 24**							
	Intercept	–0.19 (0.272)		–0.69		.48	
	Date	0 (0)		0.84		.39	
**Topic 25**							
	Intercept	0.067 (0.04)		1.52		.12	
	Date	0 (0)		–1.26		.20	

^a^Significant *P*<.05.

Multimedia Appendix 3 presents examples of texts and narratives shared by users in the Reddit r/Asthma forum, organized by each identified topic.

In addition, a clustering and correlation of all topics was observed, except topics 3, 6, and 13 (Hope of getting better soon; Use of inhaled steroids as rescue medication; and Dosage of Budesonide aerosol; respectively) referring to the use of steroids and Budesonide to accelerate the improvement of symptoms; and topics 17, 20, and 23 (Influence of respiratory symptoms such as cough; Asking for advice from people who have asthma; and Change of symptoms at times of the year when temperatures are lower; respectively) related to asking for advice from those who have already experienced asthma on the management of respiratory symptoms such as cough, especially at times of lower temperatures (Multimedia Appendix 4).

The graphs with the model predictions show how, over the year, the probability of posts containing topics 20 (Request for advice to people with asthma*) *and 22* *(Times of the year when attacks occur) appear in autumn 2024, decreasing over 2024. In the case of Topic 22, they increase again in the autumn of 2024. Conversely, Topic 21 (Medical tests to be checked periodically), increases especially in the summer-autumn of 2024 (Multimedia Appendix 5).

### Analysis of Feelings and Polarity

The proportion of feelings and emotions and scores showed a stable trend throughout the year (Multimedia Appendix 6). In addition, a nonnormal distribution in polarity was confirmed (*P*<.001).

In relation to the contrast of the hypotheses, the significant LRT indicated that the ARMA model is the one that presented the best fit, except in the NRC lexicon of emotions, which presents better results in the AR1 model. Meanwhile, in polarity, the best-fitting model was the model without autocorrelation structure (Multimedia Appendix 7).

Significant differences were confirmed in the proportion of categories appearing in each of the dictionaries used versus the reference category. In contrast, time has no significant effect on the proportion of feelings and emotions or polarity (Multimedia Appendix 8).

Sentiment and emotion analysis produced varying results depending on the lexicons applied. For instance, categorical analysis showed a higher likelihood of positive sentiments and score values of 2 using the AFINN lexicon. In contrast, the Stanford NLP, Bing, and NRC lexicons highlighted significant negative sentiment. Additionally, the NRC lexicon revealed notable associations with the emotions of anticipation, fear, and trust (Multimedia Appendix 9).

The following lines present excerpts from Reddit posts in which both negative and positive emotional expressions are identified. Each example is accompanied by the date of publication. One post describes a near-death experience due to worsening asthma symptoms:

I expressed my feelings towards the limitation that asthma brings to me…well, 2 days ago, I almost died…I had an asthma attack after minimal exercise.

Another post reflects intense frustration caused by the progression of the disease:

My asthma has been flaring up more frequently than usual. It’s stopped me from seeing friends, working, and made me lose sleep. This is causing me a significant amount of stress and anxiety, too, which I know also makes it worse.

A third example illustrates how an asthma crisis can complicate air travel:

Had an asthma attack at the beginning of a long international flight…it was really scary being trapped in that confined space, not being able to breathe.

On the other hand, positive experiences were also identified:

No doctor wanted to diagnose me as asthmatic, but the only thing that controlled my coughing attacks was corticosteroids…I am grateful to the doctor who agreed to prescribe me.

## Discussion

### Principal Findings

The analysis of Reddit’s r/Asthma forum has made it possible to identify and classify user-generated topics based on their weight and relevance. The most prominent topics include the causes of asthma and the symptoms and severity of attacks, followed by seasonal patterns of exacerbations and corticosteroid inhaler dosing. Posts display a wide range and intensity of emotions, with a noticeable tendency toward the use of negative language when discussing asthma.

### Comparison With Previous Work

The analysis of social networks (including Reddit) and their ability to influence risk behaviors, and the construction of people’s opinions, grew during the COVID-19 pandemic [44-46] because of their use as tools for obtaining and cross-checking information, sharing knowledge and as a source of help in dealing with diseases and applying strategies or treatments [47-50]. However, the use of online health information carries significant risks related to the acquisition of reliable knowledge [51-55]. To obtain trustworthy, evidence-based medical or scientific information, users must navigate through a vast amount of misinformation and poor-quality content, including posts that promote unhealthy practices or unproven treatments [52,54-56]. As a result, individuals may make decisions that could potentially harm their health [52-54]. A key factor in acquiring reliable and safe online health information is enhancing the digital literacy and search skills of lay users [57]. Moreover, several recommendations have been proposed to support safe and informed health information searches online, including (1) using trustworthy websites with official domains such as .gov, .edu, or .org; (2) verifying authorship and sources, and prioritizing content reviewed by scientific societies, health organizations, or accredited institutions; (3) avoiding personal testimonials as clinical evidence, since anecdotal narratives do not substitute scientific knowledge; and (4) consulting health care professionals before making any medical decisions [51,54,58,59].

In the field of pulmonology and respiratory diseases, the Reddit social media platform has previously been used to identify unmet needs, sentiments toward areas of interest, shared patient concerns, and the adoption of (unhealthy) behaviors related to e-cigarette use during the COVID-19 pandemic [60], lung cancer [61], vaping use [62], and respiratory lesions [63,64]. However, no previous work from Reddit’s analysis has been found for people with asthma. Asthma carries a significant burden on patients’ lives, with an important impact on their quality of life and health loss burden, measured by disability-adjusted life years, years of life lost, and years lived with disability [65]. Ten Have et al [66] showed that there are 4 asthma symptoms that, due to their impact, need to be prioritized in their resolution: fatigue, sleep disturbance, impairment of physical activity, and work-related symptoms. Moreover, our results do not show posts with relevant weight on fatigue or sleep disturbances, but on walking ability (topic 1, the fifth by weight, see Figure 1), and on work activity (topic 9, the 12th by weight). Our results revealed posts and comments from patients with Asthma on Reddit discussing the onset of asthma attacks, changes in symptoms throughout the year, and seasonal variations linked to colder temperatures (topics 2, 19, and 23). This interest aligns with previous studies showing that extreme temperatures—both hot and cold—can trigger asthma attacks and worsen symptoms [67,68].

The strategy for asthma prevention and remission of the European Forum for Research and Education in Allergy and Airway Diseases Consensus Statement [69] adopts a phenotype-centered approach aimed at improving clinical outcomes and preserving health-related quality of life. This strategy moves away from the current “one-size-fits-all” concept, which focuses on symptom-oriented treatment strategies. In addition, there is growing support among experts for including nonpharmacological and interdisciplinary interventions—such as breathing exercises and multicomponent services—in the asthma management “toolbox.” These approaches aim to help manage clinical symptoms, improve patients’ quality of life, reduce health care costs, and address unmet needs [70]. This would help to decrease the discordance between compliance rates reported by patients with asthma and physicians [71].

Additionally, another aim would be to avoid loss of adherence to treatments. Thus, Amin et al [72] described how denial of the disease and of the need for long-term treatment, along with poor physician-patient communication, suboptimal knowledge of asthma medication (lack of understanding of the distinction between maintenance and reliever inhalers), suboptimal inhaler technique, and the high cost of asthma medication, were key factors in poor medication adherence. Access to pharmacological treatment, its correct use, identification of adverse effects, and use of other therapies appear in our results in six topics (Topics 4, 6, and 13-16), with special consideration of inhaled treatments as a means of administration.

At this point, patient participation is key to treatment adherence and adoption of healthy behaviors. Kang et al [73], in their systematic review and meta-synthesis of qualitative studies on shared decision-making, highlight that patients with asthma engage in shared decision-making when they have the opportunity (ie, environmental factors such as social, cultural, and institutional conditions that either facilitate or hinder individual behavior). The capability (skills, knowledge, and abilities required for individuals to engage in a particular behavior), and the motivation (internal psychological factors that drive individuals to choose specific behaviors based on their motivations and goals. The authors of this study believe that Reddit could be a tool used by patients with asthma to gain more capacity and opportunity to manage the disease, its symptoms, and treatments.

Another possible explanation for the use of Reddit as a source of information, obtaining medications and guidelines for the use of pharmaceutical products and drugs, may be related to the price of health care provided by professionals (doctors) and the cost of drugs [72,74,75]. Zhang et al [65], in their study on health loss and the economic burden of asthma in China, described an annual direct cost of between US $348 and US $1187 per capita, indirect costs of US $7 to US $1195, and hospitalization costs of US $177 to US $1547, influenced by the frequency and severity of acute exacerbations, comorbidities, and treatment adherence. Each country has different health systems with their own models of resource organization (public vs private), which could influence access to health care, medical information and recommendations, and pharmacological treatments for people with asthma [75].

Our results revealed a range of emotions and sentiments, with notable differences between the positive sentiment scores found using the AFINN lexicon and the negative sentiments identified through the Stanford NLP, Bing, and NRC lexicons. This variability in both positive and negative emotions also appears in the study by Volpato et al [74], where patients with uncontrolled asthma experienced a wide range of emotions that impacted their daily lives. Fear and anxiety appeared alongside the unpredictability of asthma attacks, while frustration and hopelessness accompanied the symptoms [74].

### Limitations and Strengths

Among the limitations of this study is the inability to confirm an asthma diagnosis in the users who participated in the r/Asthma forum on Reddit, as well as the inability to determine other sociodemographic data of the forum participants [40]. However, the anonymous and private nature of social media would allow users to share questions, doubts, embarrassing topics, and make comments without restrictions that they would not make in other contexts [10]. Another limitation is that there was no uniformity in the comments, with some posts providing more information and content than others. This limitation was resolved by including all possible response threads derived from the posts in the study. The third limitation is that no keywords or combinations were used to search for and include posts in this study. In our study, since there were no previous studies on Reddit content related to asthma, all posts and threads generated during the study year were included. Finally, the reliability of the information shared on Reddit and other online social media platforms is essential for fostering trust in such content, promoting healthy behaviors, and protecting users’ health [54]. In this initial study conducted within the Reddit r/Asthma forum, we presented the thematic content and types of information shared by users, as well as the emotional expressions and polarity of their language. Based on this preliminary work, further research would be justified to investigate the credibility and reliability of the information shared in thematic posts, and to explore potential relationships or associations between specific emotional states and the quality of the information provided.

In relation to strengths, social media analysis (Reddit) can provide key information compared to other data collection methods, such as traditional surveys, without being limited by issues such as sensitivity or taboos surrounding certain topics [10,40]. Conversely, among all available online social media platforms, only Reddit was used due to its structure based on thematic forums or communities (subreddits), where users share content and engage in discussions focused on specific topics or interests. This structure contrasts with other platforms, such as Instagram or X, which are primarily oriented toward following individuals. Moreover, content visibility on Reddit is determined by user evaluations—either positive or negative—rather than by algorithms that prioritize virality or engagement metrics. Reddit also enables extensive discussions without restrictions on character count, image format, or source type, unlike other platforms that impose limitations based on character length (X), visual format (Instagram), or social network boundaries (Facebook). This would allow for the collection of valuable insights into users’ motivations and the nature of the content they share. Furthermore, another strength of Reddit analysis for understanding patient feelings and behaviors is that large volumes of data can be obtained in a short period of time, and user-generated content can be processed quickly [76,77]. In addition, social media generates and shares a large amount of varied information on health and disease topics, allowing patients and their families to learn about different aspects of health care, such as diagnostic processes, treatments, coping strategies, professional advice, etc.

### Conclusions

These findings can help uncover hidden needs and challenges faced daily by forum users and may assist health care professionals in developing targeted interventions aimed at improving knowledge around key topics. Identifying the most frequently shared thematic content (weighted topics) is relevant for health care professionals, as it may not reflect their clinical priorities. It also enables the assessment of information quality and the detection of potentially risky behaviors that are not supported by scientific evidence. In future research, it would be necessary to examine the scientific quality of the information shared in posts within Reddit’s r/Asthma forum and its relationship to emotional state when sharing posts.

## Appendix

Multimedia Appendix 1 Diagnostic model plots by topics number.

Multimedia Appendix 2 Best selected topic models exclusivity versus semantic coherence graph.

Multimedia Appendix 3 Examples of texts and narratives from the Reddit asthma forum by topic. Each post includes the date it was posted.

Multimedia Appendix 4 Topics correlation. Blue line thickness is proportional to correlational value.

Multimedia Appendix 5 Significant topics time-change predicted values between October 2023 and October 2024.

Multimedia Appendix 6 Reddit r/Asthma posts descriptive sentiments and emotions by dictionary and month.

Multimedia Appendix 7 Likelihood ratio test for sentiments and emotions by dictionary time series model comparison (ap values).

Multimedia Appendix 8 Final Reddit r/Asthma posts sentiments and emotions by dictionary time series models.

Multimedia Appendix 9 Significant sentiments and emotions category overall predicted values by dictionary.

## Abbreviations

AR1: autoregressive structure with lag 1
ARMA: autoregressive moving average structure
EDF: effective degrees of freedom
EQUATOR: Enhancing the Quality and Transparency of Health Research
GAMM: generalized additive mixed model
IgE: immunoglobulin E
LMM: linear mixed model
LRT: likelihood ratio test
NRC: National Research Council Canada
Stanford NLP: Stanford Natural Language Processing

## Data Availability

The datasets generated and analyzed during this study is not publicly available; although Reddit posts are publicly available and anonymized by username, Redditors did not consent for their content to be shared publicly in a curated dataset. The dataset is available from the corresponding author on reasonable request for research purposes.
