# Supplementary figures and images for: What Patients With Asthma Share When No One Listens: Multimethod Observational Study of Patient Narratives on Reddit
Source: J Med Internet Res. 2026 Jan 8;28:e77027. doi: 10.2196/77027 (PMC12828316; doi:10.2196/77027)

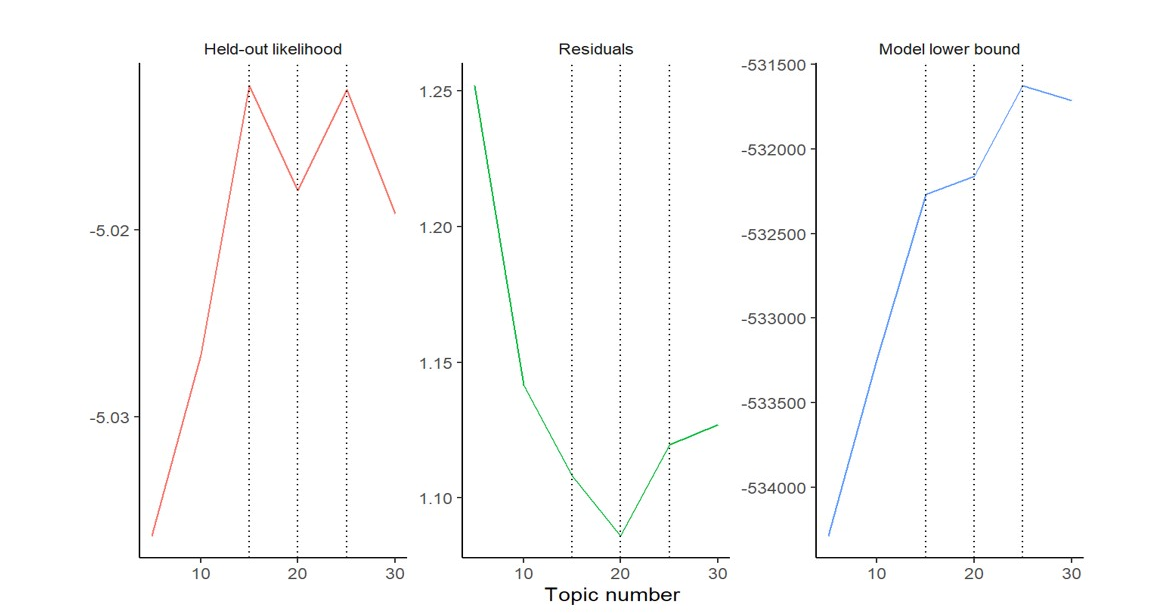

Supplement: Multimedia Appendix 1 [file jmir_v28i1e77027_app1.png]

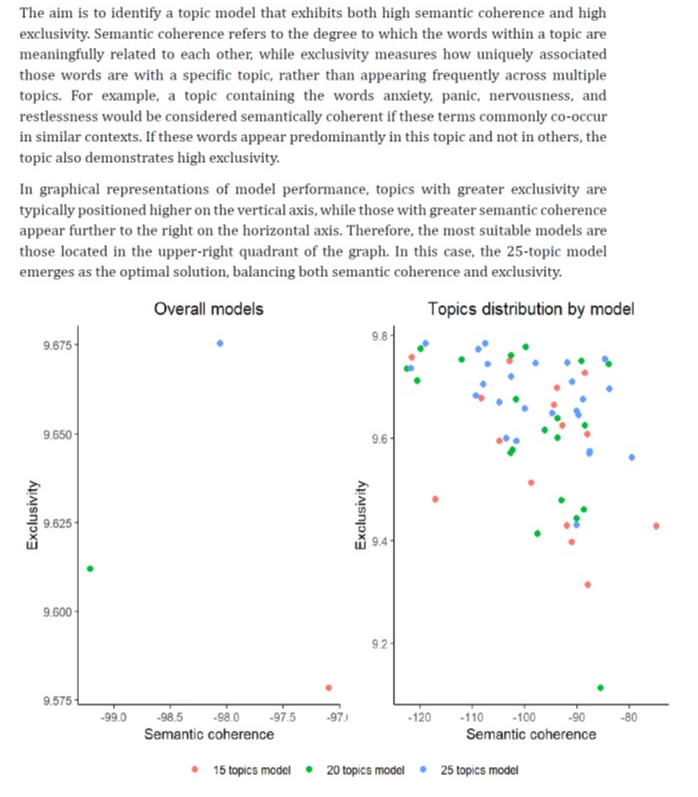

Supplement: Multimedia Appendix 2 [file jmir_v28i1e77027_app2.png]

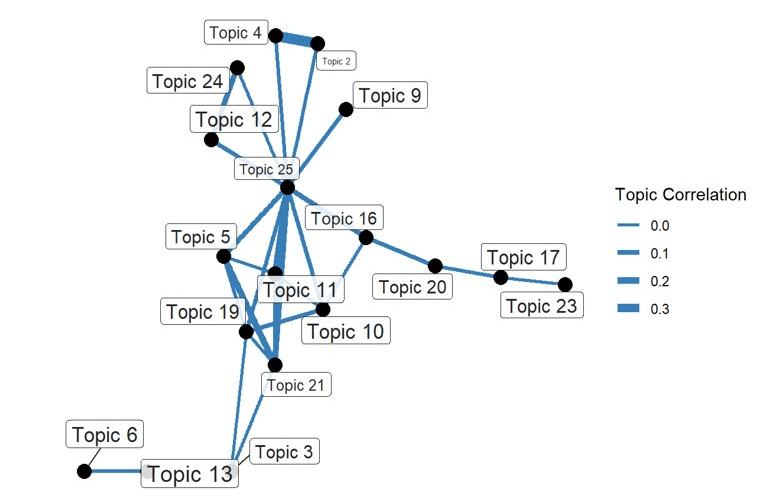

Supplement: Multimedia Appendix 4 [file jmir_v28i1e77027_app4.png]

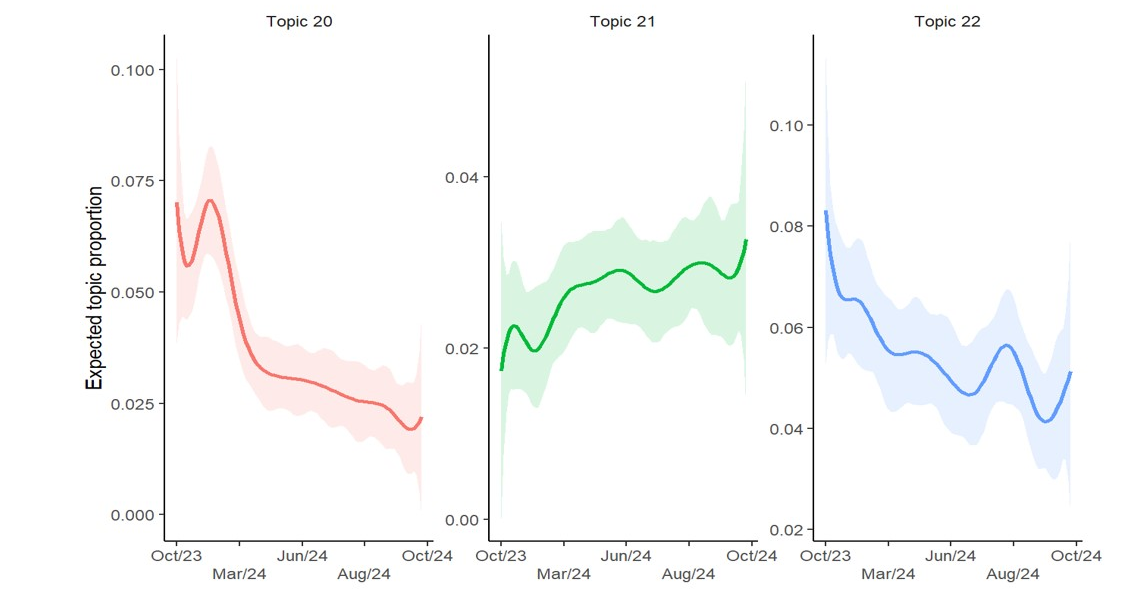

Supplement: Multimedia Appendix 5 [file jmir_v28i1e77027_app5.png]

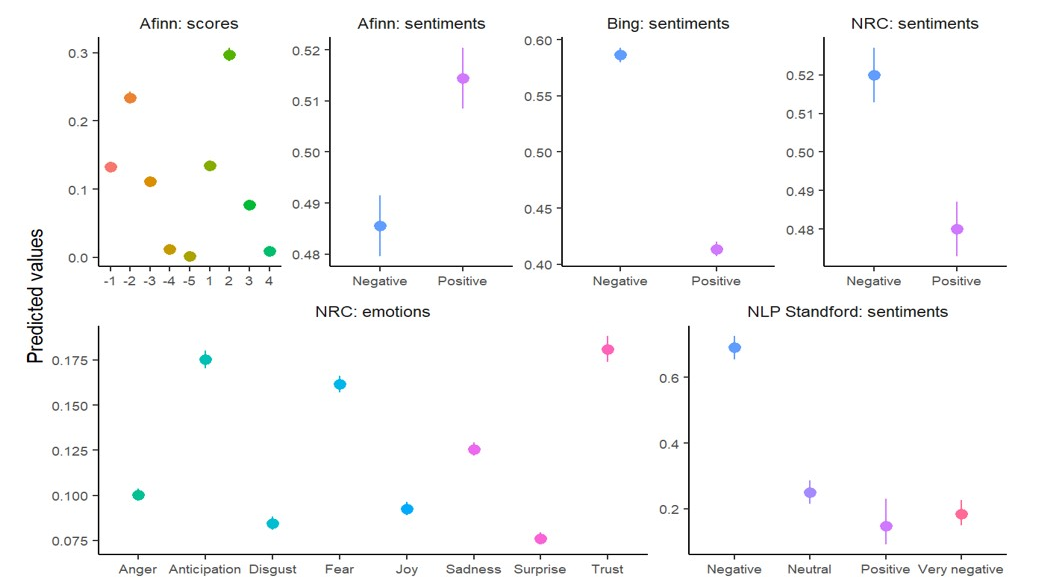

Supplement: Multimedia Appendix 9 [file jmir_v28i1e77027_app9.png]
