# Supplementary material for: What Patients With Asthma Share When No One Listens: Multimethod Observational Study of Patient Narratives on Reddit
Source: J Med Internet Res. 2026 Jan 8;28:e77027. doi: 10.2196/77027 (PMC12828316; doi:10.2196/77027)
Supplement: Multimedia Appendix 3 [file jmir_v28i1e77027_app3.docx]

**Table S1.** Examples of texts and narratives from the Reddit asthma forum by topic. Each post includes the date it was posted.

| Topic | Description | Post |
| --- | --- | --- |
| **Topic 1** | Influence of respiratory problems on the ability to walk | *“(…) if I try to walk and start talking at the same time. Instant shortness of breath. And it bad.”* |
| **Topic 2** | Timing of attacks | *“Had an allergy test today. Didn’t have any asthma issues in last 20 years. Had 3 asthma attacks in the last month.”* |
| **Topic 3** | Hopes for rapid improvement | *“My symptoms of wheezing, breathlessness, exhaustion, extreme fatigue, dizziness, came on very suddenly (…) I've been on Montelukast so around 2 months and I do feel that I've started to see results - I sleep better, and wake up more easily, I can walk around more times per week, I can finally cook for myself (…) ”* |
| **Topic 4** | Adverse effects of Prednisone that may justify stopping its use | *“(…) leave your comments of your current prescribed inhaler experiences. You can help somebody get off a bad medication …”* |
| **Topic 5** | Gratitude for improvement | *“I recently ended up in hospital because my shortness of breath got so bad one day out of nowhere, it was extremely laboured breathing (…) this new specialist bless his soul, looked at me and said 'you have asthma, lets fix you up'. He prescribed me with budesonide &amp; Formoterol inhalers on Monday (now 2 days later) and WOW. I can breath again.”* |
| **Topic 6** | Use of inhaled steroids as rescue medication | *“(…) its been better post this last round of steroids (…) I essentially had to beg the er doctor to prescribe prednisone because I suddenly got worse (…)”* |
| **Topic 7** | Symptoms and severity of asthma attacks | *“Ive had this cough for weeks, sometimes it be thru out the day, and be worse at night, its horrible during mornings, recently developed wheezing and unable to take a deep breath”* |
| **Topic 8** | Medical tests to be monitored | *“I was diagnosed with asthma (…) I started to control it properly (…) I bought a peak flow meter and started recording measurements in a mobile app.”* |
| **Topic 9** | Influence of respiratory problems on work activity | “*I have asthma (…). It doesn't affect me unless I do some heavy work or walk or run (…) in such cases and stop if stop doing anything*.” |
| **Topic 10** | Influence of respiratory problems on quality of life in general terms | *“(…) my asthma has been flaring up more frequently (…) It stopped me seeing friends, working and made me lose sleep.”* |
| **Topic 11** | Influence of tobacco | *“I quit weed vaping for 3 weeks now (…) my lungs feel 10x better. I go running with ease. I used to need my rescue almost immediately after smoking and I was taking my steroid (…)”* |
| **Topic 12** | Need for information | *“My doctor said my asthma went away to the point I didn’t need to keep an inhaler (…) But I have been feeling asthma symptoms again and I searched it and& it looks like asthma doesn’t just go away? Has anyone experienced a little remission in asthma and then had it come back? If so, did you have trouble when you went to a new doctor?”* |
| **Topic 13** | Budesonide Spray Dosage | *“ (…) he seems well controlled when we nebulize him about three times during the day (…) We are on day 6 and we were able to get by with 2 nebulizer treatments. His pulmonologist prescribed decadron because of how long it's been but should I just wait if the albuterol neb is helping to control it and see if he kicks the virus on his own?”* |
| **Topic 14** | How to access Albuterol inhalers for wheezing without medical insurance | “*Why can't you buy a rescue inhaler OTC? Like I'm dying over here with no insurance, and I absolutely cannot afford to see a doctor to get my script refilled. (…) Anyone have advice for getting one for cheap?”* |
| **Topic 15** | Hospitalization with oxygen therapy in case of acute exacerbation | *“(…) i had my first bad asthma flare, my oxygen was low but thankfully with help of the hospital and I improved (…)”* |
| **Topic 16** | Search for any asthma-related therapy | *“If you are having an asthma attack, caffeine can act as a weak bronchodilator opening your lungs (…)”* |
| **Topic 17** | Influence of respiratory symptoms such as coughing | *“… my fiance has asthma and has been struggling to sleep well due to flare ups at night where he coughs throughout the night. (…) It hard sees him wake up exhausted and him struggling to breath.”* |
| **Topic 18** | Causes of asthma | *“Deodorant and/or fabric softener are my number one trigger. And people use a lot. It seems that the world is not made for us.”* |
| **Topic 19** | Symptom change throughout the year | *“I’ve been seeing a lot of posts about medicine dosage increases (…) I was shocked when my chest felt tight this early (…) i just wanted to let you know that this year has been brutal.”* |
| **Topic 20** | Request for advice to people who have asthma | *“I live in an area that is full of trees, flowers, bushes, and grass and the air is full of pollen (…) I had a severe asthma attack (…) so I ended up calling in sick today because I needed to seek medical help (…) I’m desperate for any tips anyone can afford to help prevent my asthma.”* |
| **Topic 21** | Medical tests that should be checked periodically | *“I still have about 3 and a half more weeks before my pulmonary function test. Please if you have had one and what all was involved? How long did it take?”* |
| **Topic 22** | Times of the year when attacks occur | *“I’ve become more sensitive to weather-related asthma exacerbations. My triggers are heat, humidity, and especially the kind of humidity/pressure changes on rainy/stormy days. These are especially uncomfortable and a little scary.”* |
| **Topic 23** | Change in symptoms at times of the year when temperatures are cooler | *“The last couple days I have needed my inhaler daily. We walk in nature on our regular path that we’ve taken for almost two years now (…) I even considered Christmas bonfires and people heating their homes with wood but I haven’t been able to smell anything odd. Just looking for ideas at what potential triggers could be so I can try to avoid them in the meantime.”* |
| **Topic 24** | Need for specialized medical care | *“… (…) I went to ER due to chest tightness (…) they transferring me to the ICU after my oxygen wasn’t improving without wearing a CPAP mask. they intubated me and gave me a feeding tube for 6 days.”* |
| **Topic 25** | Gratitude for the assistance received | *“(…) the only thing that controlled my coughing attacks were corticosteroids and inhaled bronchodilators. I am eternally grateful to the doctor who agreed to prescribe me these medications.”* |
