# Supplementary material for: What Patients With Asthma Share When No One Listens: Multimethod Observational Study of Patient Narratives on Reddit
Source: J Med Internet Res. 2026 Jan 8;28:e77027. doi: 10.2196/77027 (PMC12828316; doi:10.2196/77027)
Supplement: Multimedia Appendix 6 [file jmir_v28i1e77027_app6.docx]

**Table S1.** Reddit R/Asthma posts descriptive sentiments and emotions by dictionary and month.

|  |  | **Oct-23** | **Nov-23** | **Dec-2023** | **Jan-2024** | **Feb-24** | **Mar-24** | **Apr-2024** | **May-24** | **Jun-24** | **Jul-24** | **Aug-2024** | **Sep-24** | **Oct-24** |
| --- | --- | --- | --- | --- | --- | --- | --- | --- | --- | --- | --- | --- | --- | --- |
| **NRC dictionary: sentiments** | Negative, (%) | 48.1 | 51.5 | 51.1 | 51.9 | 49.6 | 51.8 | 53.8 | 49.7 | 54.4 | 50.9 | 49.7 | 53.3 | 52.4 |
|  | Positive, (%) | 51.9 | 48.5 | 48.9 | 48.1 | 50.4 | 48.2 | 46.2 | 50.3 | 45.6 | 49.1 | 50.3 | 46.7 | 47.6 |
| **NRC dictionary: emotions** | Anger, (%) | 9.8 | 10.1 | 9.8 | 10.1 | 10.6 | 9.0 | 9.5 | 10.1 | 11.1 | 10.4 | 9.5 | 10.1 | 9.1 |
|  | Anticipation, (%) | 18.0 | 16.2 | 18.0 | 16.5 | 17.3 | 17.8 | 17.5 | 17.8 | 18.0 | 17.2 | 18.5 | 17.5 | 18.1 |
|  | Disgust, (%) | 8.9 | 10.2 | 9.6 | 7.9 | 7.1 | 9.1 | 10.3 | 7.9 | 8.3 | 7.8 | 7.5 | 8.8 | 8.7 |
|  | Fear, (%) | 14.8 | 16.7 | 14.6 | 16.4 | 16.2 | 15.8 | 15.1 | 16.4 | 17.0 | 16.2 | 16.3 | 16.6 | 15.9 |
|  | Joy, (%) | 11.7 | 9.1 | 9.9 | 9.0 | 9.3 | 10.6 | 8.6 | 9.8 | 8.8 | 9.4 | 8.8 | 8.3 | 8.7 |
|  | Sadness, (%) | 13.4 | 12.0 | 12.1 | 13.3 | 12.7 | 11.5 | 13.8 | 11.4 | 12.5 | 12.4 | 12.5 | 13.0 | 12.9 |
|  | Surprise, (%) | 6.9 | 7.4 | 7.3 | 7.3 | 7.4 | 7.9 | 8.0 | 8.0 | 7.4 | 7.9 | 7.3 | 7.6 | 7.2 |
|  | Trust, (%) | 16.5 | 18.4 | 18.8 | 19.7 | 19.4 | 18.2 | 17.1 | 18.7 | 16.9 | 18.7 | 19.5 | 18.2 | 19.3 |
| **Afinn dictionary: sentiments** | Negative, (%) | 44.2 | 45.5 | 48.4 | 49.5 | 46.5 | 44.8 | 49.2 | 46.0 | 50.5 | 49.6 | 46.8 | 49.7 | 49.2 |
|  | Positive, (%) | 55.8 | 54.5 | 51.6 | 50.5 | 53.5 | 55.2 | 50.8 | 54.0 | 49.5 | 50.4 | 53.2 | 50.3 | 50.8 |
| **Afinn dictionary: scores** | -1, (%) | 6.9 | 13.2 | 13.6 | 12.3 | 12.7 | 11.4 | 13.3 | 13.9 | 13.9 | 13.4 | 11.9 | 14.2 | 14.3 |
|  | -2, (%) | 21.5 | 21.4 | 23.4 | 25.8 | 22.6 | 22.7 | 21.5 | 21.4 | 24.3 | 22.8 | 22.6 | 24.4 | 24.7 |
|  | -3, (%) | 15.0 | 10.0 | 10.3 | 9.9 | 10.0 | 9.8 | 12.5 | 10.0 | 11.2 | 11.9 | 11.4 | 10.7 | 9.5 |
|  | -4, (%) | 0.9 | 0.9 | 1.0 | 1.3 | 1.0 | 0.9 | 1.9 | 0.8 | 1.0 | 1.5 | 0.7 | 0.4 | 0.6 |
|  | -5, (%) | 0.0 | 0.1 | 0.1 | 0.1 | 0.1 | 0.0 | 0.1 | 0.0 | 0.1 | 0.0 | 0.1 | 0.1 | 0.1 |
|  | 1, (%) | 13.3 | 12.8 | 14.6 | 13.7 | 15.4 | 13.4 | 11.9 | 13.0 | 14.6 | 12.7 | 13.2 | 13.6 | 14.4 |
|  | 2, (%) | 31.3 | 33.5 | 28.6 | 28.3 | 28.9 | 32.4 | 30.2 | 32.7 | 26.5 | 29.0 | 30.6 | 29.6 | 29.5 |
|  | 3, (%) | 10.3 | 7.0 | 7.2 | 7.6 | 8.1 | 8.1 | 7.6 | 7.6 | 7.5 | 7.9 | 8.5 | 6.4 | 6.2 |
|  | 4, (%) | 0.9 | 1.2 | 1.2 | 0.9 | 1.0 | 1.3 | 1.0 | 0.7 | 0.9 | 0.8 | 1.0 | 0.7 | 0.7 |
| **Bing dictionary: sentiments** | Negative, (%) | 55.6 | 56.2 | 60.2 | 59.5 | 56.6 | 55.6 | 56.7 | 56.9 | 60.5 | 60.6 | 57.0 | 60.5 | 59.1 |
|  | Positive, (%) | 44.4 | 43.8 | 39.8 | 40.5 | 43.4 | 44.4 | 43.3 | 43.1 | 39.5 | 39.4 | 43.0 | 39.5 | 40.9 |
| **NLP Standford dictionary: sentiments** | Negative, (%) | 100.0 | 70.0 | 74.1 | 66.7 | 59.3 | 67.7 | 69.0 | 73.3 | 66.7 | 70.0 | 66.7 | 80.0 | 63.3 |
|  | Neutral, (%) | 0.0 | 23.3 | 18.5 | 20.0 | 22.2 | 25.8 | 13.8 | 20.0 | 20.0 | 20.0 | 20.0 | 13.3 | 30.0 |
|  | Positive, (%) | 0.0 | 0.0 | 0.0 | 3.3 | 0.0 | 0.0 | 3.4 | 0.0 | 3.3 | 3.3 | 3.3 | 6.7 | 3.3 |
|  | Very negative, (%) | 0.0 | 6.7 | 7.4 | 10.0 | 18.5 | 6.5 | 13.8 | 6.7 | 10.0 | 6.7 | 10.0 | 0.0 | 3.3 |
| **Polarity scores** |  | -0.05 [-0.22, 0.18] | 0.00 [-0.20, 0.17] | -0.05 [-0.25, 0.11] | 0.00 [-0.17, 0.14] | 0.00 [-0.21, 0.13] | 0.00 [-0.18, 0.15] | 0.00 [-0.21, 0.13] | 0.00 [-0.16, 0.15] | 0.00 [-0.20, 0.14] | 0.00 [-0.23, 0.13] | 0.00 [-0.20, 0.17] | 0.00 [-0.24, 0.13] | 0.00 [-0.21, 0.13] |

Data expressed with median [interquartile range] or with relative values (%)
