# Supplementary material for: What Patients With Asthma Share When No One Listens: Multimethod Observational Study of Patient Narratives on Reddit
Source: J Med Internet Res. 2026 Jan 8;28:e77027. doi: 10.2196/77027 (PMC12828316; doi:10.2196/77027)
Supplement: Multimedia Appendix 7 [file jmir_v28i1e77027_app7.docx]

**Table S3.** Likelihood ratio test for sentiments and emotions by dictionary time series model comparison (^a^*P* values).

| **Afinn dictionary: sentiments** | AR model | X^2^(2, 8) =13.84, *P*=.001 | ARMA model | X^2^(4, 12) =26.957, *P*<.001 |
| --- | --- | --- | --- | --- |
|  | ARMA model | X^2^(4, 12) =26.957, *P*<.00 |  |  |
| **Afinn ditionary: scores** | AR1 model | X^2^(9, 29) =1099.631, *P*<.00 | ARMA model | X^2^(1, 30) =6.658, *P*=.01 |
|  | ARMA model | X^2^(1, 30) =6.658, *P*=0.01 |  |  |
| **Bing dictionary: sentiments** | AR1 model | X^2^(2, 8) =185.391, *P*<.001 | ARMA model | X^2^(4, 12) =15.965, *P*=.00 |
|  | ARMA model | X^2^(4, 12) =15.965, *P*=.003 |  |  |
| **NRC dictionary: emotions** | AR1 model | X^2^(8, 26) =923.191, *P*<.001 | ARMA model | X^2^(2, 28) =0.702, *P*=.70 |
|  | ARMA model | X^2^(2, 28) =0.702, *P*=.704 |  |  |
| **NRC dictionary: sentiments** | AR1 model | X^2^(2, 8) =27.425, *P*<.001 | ARMA model | X^2^(2, 10) =10.193, *P*=.006 |
|  | ARMA model | X^2^(2, 10) =10.193, *P*=.006 |  |  |
| **Polarity sores** | AR1 model | X^2^(1, 5) =0.016, *P*=.90 | ARMA model | X^2^(1, 6) =0.002, *P*=.96 |
|  | ARMA model | X^2^(1, 6) =0.002, *P*=.96 |  |  |
| **NLP Standford dictionary: sentiments** | AR1 model | X^2^(4, 14) =140.802, *P*<.001 | ARMA model | X^2^(0, 14) =0.045, *P*<.001**^c^** |
|  | ARMA model | X^2^(0, 14) =0.045, *P*<.001 |  |  |

AR1: Auto-regresive correlation structure with lag 1; ARMA: Auto-regresive moving average correlation structure; X^2^: Chi squared statistic; Between parenthesis: Degrees of freedom; ^a^significant p<0.05
