# Supplementary material for: What Patients With Asthma Share When No One Listens: Multimethod Observational Study of Patient Narratives on Reddit
Source: J Med Internet Res. 2026 Jan 8;28:e77027. doi: 10.2196/77027 (PMC12828316; doi:10.2196/77027)
Supplement: Multimedia Appendix 8 [file jmir_v28i1e77027_app8.docx]

**Table S1.** Final Reddit r/Asthma posts sentiments and emotions by dictionary time series models.

|  |  | Coefficient (SE), t(df) or Z, *P* valule^a^ |  |  | Coefficient (SE), t(df) or Z, *P* valule^a^ |
| --- | --- | --- | --- | --- | --- |
| **NRC dictionary: sentiments** | (Intercept) | 0.08 (SE**^a^**=0.014), t (102) =5.519, *P*<.001 | **Afinn dictionary: sentiments** | (Intercept) | -0.058(SE=0.012), t (102) = -4.782, *P*<.001 |
|  | Positive vs. negative | -0.16(SE=0.02), t (102) =-7.805, *P*<.001 |  | Positive vs. negative | 0.116(SE=0.017), t (102) = 6.762, *P*<.001 |
|  | Date: Negative | 0.012(SE=0.036), t (102) =0.346, *P*=.73 |  | Date: Negative | -0.038(SE=0.031), t (102) = -1.219, *P*=.22 |
|  | Date: Positive | -0.012(SE=0.036), t (102) =-0.346, *P*=.73 |  | Date: Positive | 0.038(SE=0.031), t (102) = 1.219, *P*=.22 |
| **NRC dictionary: emotions** | (Intercept) | -2.197 (SE=0.017), Z=-127.914, *P*<.001 | **Afinn dictionary: scores** | (Intercept) | -1.876 (SE=0.024), Z=-79.554, *P*<.001 |
|  | Anticipation vs. anger | 0.647 (SE=0.024), Z=26.671, *P*<.001 |  | -2 vs. -1 | 0.692 (SE=0.034), Z=20.53, *P*<.001 |
|  | Disgust vs. anger | -0.166 (SE=0.024), Z=-6.836, *P*<.001 |  | -3 vs. -1 | -0.248 (SE=0.034), Z=-7.312, *P*<.001 |
|  | Fear vs. anger | 0.549 (SE=0.024), Z=22.663, *P*<.001 |  | -4 vs. -1 | -2.602 (SE=0.035), Z=-73.825, *P*<.001 |
|  | Joy vs. anger | -0.096 (SE=0.024), Z=-3.94, *P*<.001 |  | -5 vs. -1 | -4.353 (SE=0.056), Z=-77.229, *P*<.001 |
|  | Sadness vs. anger | 0.256 (SE=0.024), Z=10.562, *P*<.001 |  | 1 vs. -1 | 0.018 (SE=0.034), Z=0.547, *P*=.58 |
|  | Surprise vs. anger | -0.318 (SE=0.024), Z=-13.095, *P*<.001 |  | 2 vs. -1 | 1.016 (SE=0.034), Z=30.12, *P*<.001 |
|  | Trust vs. anger | 0.714 (SE=0.024), Z=29.479, *P*<.001 |  | 3 vs. -1 | -0.659 (SE=0.034), Z=-19.374, *P*<.001 |
|  | Date: anger (spline 1) | -0.014 (SE=0.026), Z=-0.54, *P*=.58 |  | 4 vs. -1 | -2.785 (SE=0.034), Z=-81.125, *P*<.001 |
|  | Date: anger (spline 2) | -0.035 (SE=0.036), Z=-0.984, *P*=.32 |  | Date: -1 (spline 1) | 0.031 (SE=0.029), Z=1.078, *P*=.28 |
|  | Date: anticipation (spline 1) | 0.034 (SE=0.02), Z=1.672, *P=*.09 |  | Date: -1 (spline 2) | 0.054 (SE=0.05), Z=1.078, *P*=.28 |
|  | Date: anticipation (spline 2) | 0.059 (SE=0.035), Z=1.672, *P*=0.09 |  | Date: -2 (spline 1) | 0.016 (SE=0.029), Z=0.537, *P*=.59 |
|  | Date: disgust (spline 1) | -0.105 (SE=0.042), Z=-2.51, *P*=.012 |  | Date: -2 (spline 2) | 0.028 (SE=0.051), Z=0.537, *P*=.59 |
|  | Date: disgust (spline 2) | -0.083 (SE=0.039), Z=-2.12, *P*=.035 |  | Date: -3 (spline 1) | 0.099 (SE=0.061), Z=1.614, *P*=.10 |
|  | Date: fear (spline 1) | 0.014 (SE=0.02), Z=0.686, *P*=.49 |  | Date: -3 (spline 2) | -0.032 (SE=0.079), Z=-0.406, *P*=.68 |
|  | Date: fear (spline 2) | 0.024 (SE=0.035), Z=0.686, *P*=.49 |  | Date: -4 (spline 1) | -0.13 (SE=0.074), Z=-1.757, *P*=.08 |
|  | Date: joy (spline 1) | -0.017 (SE=0.037), Z=-0.465, *P*=.64 |  | Date: -4 (spline 2) | -0.484 (SE=0.094), Z=-5.176, *P*<.001 |
|  | Date: joy (spline 2) | -0.082 (SE=0.037), Z=-2.185, *P*=.029 |  | Date: -5 (spline 1) | -0.144 (SE=0.059), Z=-2.429, *P*=.016 |
|  | Date: sadness (spline 1) | 0.015 (SE=0.02), Z=0.754, *P*=.45 |  | Date: -5 (spline 2) | -0.251 (SE=0.103), Z=-2.429, *P*=.016 |
|  | Date:sadness (spline 2) | 0.026 (SE=0.035), Z=0.754, *P*=.45 |  | Date:1 (spline 1) | -0.005 (SE=0.03), Z=-0.164, *P*=.87 |
|  | Date: surprise (spline 1) | 0.046 (SE=0.042), Z=1.093, *P*=.27 |  | Date:1 (spline 2) | -0.008 (SE=0.052), Z=-0.164, *P*=.87 |
|  | Date: surprise (spline 2) | -0.018 (SE=0.039), Z=-0.473, *P*=.63 |  | Date:2 (spline 1) | -0.006 (SE=0.029), Z=-0.199, *P*=.84 |
|  | Date: trust (spline 1) | -0.051 (SE=0.044), Z=-1.179, *P*=.23 |  | Date:2 (spline 2) | -0.01 (SE=0.051), Z=-0.199, *P*=.84 |
|  | Date: trust (spline 2) | 0.049 (SE=0.039), Z=1.262, *P*=.20 |  | Date:3 (spline 1) | 0.083 (SE=0.065), Z=1.27, *P=*.20 |
| **Bing dictionary: sentiments** | (Intercept) | 0.349(SE=0.013), t (102) =25.866, *P*<.001 |  | Date:3 (spline 2) | -0.118 (SE=0.087), Z=-1.359, *P*=.17 |
|  | Positive vs. negative | -0.698(SE=0.019), t (102) =-36.58, *P*<.001 |  | Date:4 (spline 1) | -0.212 (SE=0.03), Z=-7.046, *P*<.001 |
|  | Date: Negative | -0.034(SE=0.034), t (102) =-0.988, *P*=.32 |  | Date:4 (spline 2) | -0.37 (SE=0.053), Z=-7.046, *P*<.001 |
|  | Date: Positive | 0.034(SE=0.034), t (102) =0.988, *P*=.32 | **Polarity scores** | (Intercept) | 0.08(SE=0.014), t (102) =5.519, *P*<.001 |
| **NLP Standford dictionary: sentiments** | (Intercept) | 0.864(SE=0.047), t (99.5) =16.375, *P*<.001 |  | Positive vs. negative | -0.16(SE=0.02), t (102) =-7.805, *P*<.001 |
|  | Neutral vs. negative | -1.652(SE=0.134), t (99.5) =-10.88, *P*<.001 |  | Date: Negative | 0.012(SE=0.036), t (102) =0.346, *P*=.73 |
|  | Positive vs. negative | -2.168(SE=0.574), t (99.5) =-3.614, *P*<.001 |  | Date: Positive | -0.012(SE=0.036), t (102) =-0.346, *P*=.73 |
|  | Very negative vs. negative | -1.865(SE=0.243), t (99.5) =-7.415, *P*<.001 |  |  |  |
|  | Date: Negative | -0.084(SE=0.189), t (99.5) =-0.386, *P*=.35 |  |  |  |
|  | Date: Neutral | -0.091(SE=0.319), t (99.5) =-0.288, *P*=.49 |  |  |  |
|  | Date: Positive | 0.027(SE=1.292), t (99.5) =0.013, *P*=.71 |  |  |  |
|  | Date: Very Negative | -0.294(SE=0.654), t (99.5) =-0.414, *P*=.48 |  |  |  |

SE: Standard error; t: t statistic; df: Degrees of freedom; Z: Wald type statistic; Spline: in Geeralized aditive models (GAMs), number of basis functions to model the time series shape; ^a^significant *P*<.05.
